# Association of *ERBB4* and *SHBG* gene polymorphisms with polycystic ovarian syndrome in South Indian women: a case–control genetic analysis

**DOI:** 10.1080/07853890.2026.2688583

**Published:** 2026-06-18

**Authors:** Sheena Mariam Thomas, Nithya Ajay, Vijayalakshmi Kandasamy, Ramakrishnan Veerabathiran

**Affiliations:** ^a^ Human Cytogenetics and Genomics Laboratory, Faculty of Allied Health Sciences, Chettinad Hospital and Research Institute, Chettinad Academy of Research and Education, Kelambakkam, Tamil Nadu, India; ^b^ Department of Obstetrics and Gynecology, Chettinad Hospital and Research Institute, Chettinad Academy of Research and Education, Kelambakkam, Tamil Nadu, India

**Keywords:** Polycystic ovarian syndrome, *ERBB4* gene, *SHBG* gene, polymorphisms, association and South Indian population

## Abstract

**Introduction:**

Polycystic ovary syndrome (PCOS) is a multifactorial endocrinological disorder with a substantial genetic component. However, the role of genes involved in follicular development and androgen regulation remains incompletely understood, particularly in South Indian populations. This study aimed to evaluate how variations in the *ERBB4* and *SHBG* genes affect PCOS risk.

**Methodology:**

A hospital–based case–control study was conducted among 400 South Indian women, comprising 200 women with PCOS and 200 age-matched healthy controls. Genomic DNA was extracted to study SNPs at *ERBB4* (rs2178575 and rs1351592) and *SHBG* (rs1799941 and rs727428) using ARMS-PCR genotyping. The study compared genotype and allele frequencies between cases and controls while assessing their associations with allelic, homozygous, heterozygous, dominant, recessive, and over-dominant genetic models. Genotyping accuracy was confirmed by re-genotyping and Sanger sequencing of a subset of samples.

**Results:**

The *ERBB4* rs2178575 polymorphism demonstrated a significant association with PCOS, as the AA genotype and A allele combination increased risk across all three genetic models, including homozygous, recessive, and allelic models. The *ERBB4* rs1351592 variant was associated with 3-fold higher risk of PCOS in heterozygous and GC carriers. The *SHBG* rs1799941 polymorphism showed a significant link to PCOS through its effects on heterozygous and allelic states, whereas rs727428 displayed no significant connection due to its monomorphic distribution.

**Conclusion:**

These findings suggest that polymorphisms in *ERBB4* and *SHBG* may contribute to PCOS susceptibility in South Indian women in a locus- and model-specific manner, revealing the intricate genetic structure that defines this medical condition.

## 1. Introduction

Polycystic Ovarian Syndrome, which people commonly call PCOS, represents the most widespread endocrine disorder because it specifically affects women who are in their reproductive years. The studies that examined different populations reported the prevalence rates of PCOS to be 8-13% among women and 6% among girls who were undergoing puberty [1,2]. The main clinical causes of irregular menstrual cycles, hirsutism, and anovulatory infertility in women stem from PCOS according to medical experts [3,4].

Women who have PCOS experience psychological symptoms because they develop anxiety and depression, and body image problems [5–7]. Women with PCOS experience common metabolic disorders, which include type 2 diabetes mellitus, insulin resistance, metabolic syndrome, obesity, prediabetes, and cardiovascular risk factors such as dyslipidemia and hypertension. PCOS causes multiple health problems, which include endometrial carcinoma, sleep apnea, and various pregnancy complications, including preeclampsia, gestational diabetes, postpartum hemorrhage, pregnancy-induced hypertension, infections, shoulder dystocia, meconium aspiration, stillbirth, operative deliveries, preterm birth, and other conditions [8]. PCOS causes reproductive problems, which diminish women’s body health as well as sexual health and their total well-being according to research findings [9]. PCOS remains a complex and mysterious disorder that doctors find difficult to diagnose and treat because its symptoms change with ageing, and doctors need to customize treatment plans according to individual patient requirements [10].

There is strong evidence that genetic influences are very significant in the development of PCOS. A heritability of 0.79 was estimated based on the Dutch twin study, thus implying a strong genetic contribution [11]. Further family studies have shown the genetic impact on PCOS, along with its symptoms, such as hyperandrogenism and insulin resistance. At present, genetics research is mainly concerned with the analysis of candidate genes, which is made possible through the advances brought by the HapMap project and other genome-wide studies. The candidate genes that have been identified are mainly those that act in the pathways of steroid hormone production, gonadotropin regulation, follicle maturation, energy expenditure, and insulin signalling [12].

The *ERBB4* gene encodes ErbB4 (HER4), an EGFR family receptor tyrosine kinase that regulates critical cellular processes including proliferation, differentiation, and survival. In the ovarian context, *ERBB4* is involved in follicular development and oocyte–somatic cell communication. Genetic variations in *ERBB4* may therefore disrupt the ovarian microenvironment, contributing to impaired folliculogenesis and the anovulatory phenotype characteristic of PCOS [13]. The genetic link to PCOS has been confirmed through multiple GWAS studies, which found *ERBB4* to be a susceptibility locus across different ethnic groups [14].

The existing research does not provide sufficient proof that *SHBG* gene polymorphisms directly connect to PCOS. Sex hormone–binding globulin (*SHBG*) functions as a plasma glycoprotein that the liver produces to control the availability of circulating sex steroids. In women with PCOS, SHBG levels are frequently reduced, mainly as a consequence of hyperinsulinemia, which suppresses hepatic SHBG production. This reduction leads to increased concentrations of free androgens and contributes significantly to the hyperandrogenic state observed in PCOS [15,16].

Though there is mounting evidence underpinning the genetic pattern of polycystic ovary syndrome, the part played by particular gene variations tied to follicular development and androgen control is still not fully grasped, especially in the case of South Indian women. *ERBB4* has been assigned a crucial role in ovarian folliculogenesis, and genome-wide association studies have identified it as a susceptibility locus for PCOS, while SHBG is recognized for its primary role in controlling the amount of androgen available in circulation. Thus, the current study intends to explore the polymorphisms of *ERBB4* and *SHBG* genes in relation to the incidence of PCOS in South Indian women with the aim of clarifying the potential influence of the genes in the susceptibility to the condition and the associated clinical manifestations. The outcomes of this research could provide better insights into the genetic makeup of PCOS in this particular group and facilitate the formulation of diagnostic and treatment methods specific to the population.

## 2. Methodology

### 2.1. Study framework and participants

This hospital-based case–control genetic association study included a total of 400 South Indian women, aged 20–35 years, comprising 200 women diagnosed with PCOS and 200 healthy controls. The study was conducted between 2024 and 2025 after obtaining ethical approval from the Institutional Human Research Ethics Committee, Chettinad Academy of Research and Education, Tamil Nadu, India (Ref. No: IHEC-II/0544/24). Written informed consent was obtained from all participants before enrollment. Participants were informed about the study objectives, procedures, potential risks, and their right to withdraw at any time without any consequences.

Participants for the study were selected from the Department of Obstetrics and Gynecology at Chettinad Hospital and Research Institute (CHRI), Kelambakkam, Tamil Nadu, and another tertiary care centre in South India. Cases of PCOS were selected from both centres, whereas the control subjects were recruited only from Chettinad Hospital. Participants were recruited in a single study period only. The same criteria of inclusion and exclusion were maintained in all recruitment centres to maintain comparability between the groups. Stratification was avoided among the participants recruited in the study by recruiting unrelated individuals of South Indian origin.

### 2.2. Diagnostic criteria and selection of participants

#### 2.2.1. PCOS cases

PCOS diagnosis was established based on the Rotterdam 2003 criteria, as recommended by the European Society of Human Reproduction and Embryology (ESHRE) and the American Society for Reproductive Medicine (ASRM), requiring the presence of at least two of the following three features. These criteria remain widely used in genetic and epidemiological studies, allowing comparability with previously published data despite the availability of updated guidelines.

1. Oligo/anovulation, defined as fewer than eight menstrual cycles per year;
2. Hyperandrogenism, assessed either clinically (modified Ferriman–Gallwey score ≥3, appropriate for Asian women) or biochemically (total testosterone ≥67 ng/dL or free testosterone ≥0.84 ng/dL);
3. Polycystic ovarian morphology (PCOM) on ultrasonography is defined as ≥12 follicles measuring 2–9 mm in diameter and/or ovarian volume >10 cm³.

Women with hyperprolactinemia, thyroid dysfunction, congenital adrenal hyperplasia, Cushing’s syndrome, or androgen-secreting tumours were excluded following clinical and biochemical evaluation. Participants who had received hormonal therapy within the preceding three months were also excluded.

#### 2.2.2. Control group

Controls included healthy females who had been sourced from the same clinic environment; they showed regular menstrual cycles, no signs of clinical or biochemical hyperandrogenism, normal ovarian structures on ultrasound assessment, and no personal or familial background of PCOS or infertility. The selection of controls was based on the same clinical and biochemical screening procedures used for case subjects, thus ensuring that no subclinical features of PCOS existed among them.

### 2.3. Clinical and biochemical assessment

The measurements of the anthropometric dimensions were performed with the help of standardized tools, and the body mass index (BMI) was computed using the formula (kg)/height (m^2^). Venous blood samples from the participants following an overnight fast were taken on the 2nd to 5th day of the early follicular phase. After being separated by centrifugation, the serum was kept at −20 °C until the time of analysis.

The hormonal profiling was done using the testing of thyroid function, where measurements of triiodothyronine (T3), thyroxine (T4) and thyroid-stimulating hormone (TSH) were performed; and the measurements of the reproductive hormones, where follicle-stimulating hormone (FSH), luteinizing hormone (LH), and prolactin were measured. All the assays were carried out through the chemiluminescent immunoassay (CLIA) method on an automated analyzer according to the manufacturer’s guidelines, with internal quality controls maintained throughout the process. After following the manufacturer’s protocol for a CLIA (chemiluminescent immunoassay) based automated laboratory method, total testosterone levels were measured. Laboratory reference ranges were used to assess biochemical hyperandrogenism; total testosterone ≥67 ng/dL or free testosterone ≥0.84 ng/dL were both considered elevated. The presence of hyperprolactinemia and/or thyroid dysfunction was ruled out by clinical assessments and by using specific laboratory-defined reference ranges. Cases of PCOS that had mild elevations of testosterone, although within the range of clinically acceptable values, were thought to be indicative of the usual endocrine disturbances associated with PCOS and did not disqualify these individuals from the study. Biochemical hyperandrogenism was defined beased on the established laboratory reference ranges, and all assays were performed under standardized conditions with appropriate quality control measures.

### 2.4. Genomic DNA extraction and quality assessment

For genetic analysis, approximately 2-5mL of peripheral blood was collected from each participant in EDTA-coated vacutainers. The salting-out method (Miller’s method) was employed to isolate genomic DNA from peripheral blood leukocytes. Using a NanoDrop 2000 spectrophotometer (Thermo Scientific), DNA concentration and purity were measured, and only samples with adequate purity (A260/A280 ratio of 1.8–2.0) were retained and stored at −20 °C for subsequent analysis.

### 2.5. SNP selection and genotyping

Based on earlier GWAS evidence, reported associations with PCOS-related characteristics, and the minor allele frequency in South Asian populations, SNPs in the *ERBB4* and *SHBG* genes were chosen, also considering the potential for functional relevance.

The ARMS-PCR method was used for genotyping purposes. PCR amplification reaction was done in a final reaction volume of 10 µL containing approximately 50-100ng of genomic DNA, 5 pmol of each primer, 200 µM dNTPs, 1.5 mM MgCl_2_, 1× reaction buffer and 0.5 U Taq DNA polymerase. Amplification was carried out using a thermal cycler (Bio-Rad T100 Thermal Cycler) under the following conditions: initial denaturation at 95 °C for 5 min, followed by 30–35 cycles of denaturation at 94 °C for 45 s, annealing at 58 °C–65 °C for 45 s–1 min (depending on primer set), extension at 72 °C for 45 s, and final extension at 72 °C for 5 min. Ethidium bromide staining was used to evaluate the amplified products, which were then analyzed through 2% agarose gel electrophoresis and UV light visualization. Genotypes were identified through the analysis of allele-specific band patterns.

### 2.6. Genotyping validation and quality control

An arbitrary selection of around 10% of the samples was re-genotyped in order to ensure the accuracy of genotyping, and this resulted in a complete agreement rate of 100%. Besides this, Sanger sequencing was used to validate the PCR products using a subset of samples from both controls and cases for each SNP, including representative genotypes where available. The chromatograms of the sequences were analyzed *via* Chromas software, and the alignment was done through BLAST to check the target polymorphisms.

### 2.7. Statistical analysis

Statistical analyses were performed using SPSS software (version 31.0.2.0 (126)). Continuous variables were expressed as mean ± standard deviation, and categorical variables as frequencies and percentages. Differences between cases and controls were evaluated using Student’s t-test or Chi-square test, as appropriate. Genotype and allele frequencies were calculated by direct counting. Hardy–Weinberg equilibrium (HWE) was assessed in the control group using the Chi-square test. The Minor allele frequencies (MAFs) for both case and control groups were calculated using the genotype counts. In order to assess the potential population stratification, the control group MAFs were compared with South Asian population data from the 1000 Genomes Project. The comparisons were carried out to evaluate the representativeness of the study population by extracting the reference allele frequencies from population-specific datasets. Associations between *ERBB4* and *SHBG* polymorphisms and PCOS risk were evaluated using unadjusted logistic regression models, and odds ratios (ORs) with 95% confidence intervals (CIs) under dominant, recessive, and additive genetic models were reported. A *p*-value <0.05 is considered to be statistically significant. Bonferroni correction was applied to account for multiple comparisons, and a corrected significance threshold of *p* < 0.002 was considered. The precision of the estimated associations was interpreted based on 95% CI rather than post hoc power calculations. Given the sample size and the observed allele frequencies, the study was adequately powered to detect moderate genetic effects, whereas smaller associations may not have been identified.

## 3. Results

### 3.1. Clinical, anthropometric, and hormonal profile of enrolled subjects

The study included 400 subjects in total, with 200 PCOS patients and 200 age-matched healthy women. The research studied people who were between 20 and 35 years old. Women with PCOS had an average age of 27.85 years, with a standard deviation of 3.70 years, whereas the control group’s average age was 27.72 years with a standard deviation of 4.40 years. The anthropometric analysis showed that PCOS patients had much higher body weight than the control group. The PCOS group showed higher BMI levels than the control group because of increased rates of overweight and obesity. The two groups displayed identical height measurements according to the height test results.

The biochemical indicators were also subjected to comparative analysis between the groups, in which thyroid hormone profiles (TSH) were substantially increased among PCOS patients relative to controls, which shows a trend toward thyroid dysfunction in women with PCOS. PCOS patients showed extreme reproductive hormonal disturbances that affected their sexual development. The PCOS group exhibited elevated FSH and LH levels, which showed the typical hormonal abnormalities that define their medical condition. The PCOS group showed significant increases in serum prolactin levels, which produced a statistically significant difference with *p* < 0.001. PCOS patients showed elevated prolactin and TSH levels despite having excluded obvious hyperprolactinemic women and women with thyroid dysfunctions from study enrollment as part of the recruitment process. The presence of mild prolactin and TSH elevations further supports reports of hormonal abnormalities being prevalent in women with PCOS. The PCOS patients showed higher total testosterone levels, which confirmed their biochemical hyperandrogenism condition. Significant differences were observed for several anthropometric and biochemical parameters, including weight, BMI, T3, T4, TSH, FSH, LH, prolactin and testosterone levels, whereas age and height did not differ significantly between the groups. The study results established that these specific variables effectively distinguished different PCOS groups and are depicted in Table 1.

**Table 1.** Comparison of anthropometric and biochemical parameters between the PCOS patients and healthy controls.

	Control Group (*n* = 200)	PCOS Group (*n* = 200)	*p*-value
**Anthropometric Factors **			
Age	27.72 ± 4.402	27.85 ± 3.70352	0.749
Weight	60.03 ± 5.769	71.1265 ± 11.16758	<0.0001
Height	159.79 ± 5.889	159.7025 ± 6.3874	0.887
BMI	23.63 ± 3.009	27.9895 ± 4.74473	<0.0001
**Biochemical Factors**			
T3 (pg/ml)	3.26 ± 0.557	2.6753 ± 0.81499	<0.0001
T4 (ng/dl)	1.42 ± 0.369	1.3252 ± 0.3955	0.014
TSH (ulU/ml)	2.12 ± 1.004	2.5528 ± 0.70693	<0.0001
FSH (mlU/ml)	6.24 ± 1.997	6.9299 ± 2.39623	0.0019
LH (mlU/ml)	6.35 ± 2.427	12.7492 ± 3.14177	<0.0001
Prolactin (ng/ml)	13.35 ± 5.529	31.9688 ± 7.73605	<0.0001
Testosterone	42.76 ± 14.587	72.7745 ± 5.61984	<0.0001

Data are presented as mean ± standard deviation (SD). *p*-values are calculated using independent sample Student’s t-test. Statistical significance was considered at *p* < 0.05.

### 3.2. Frequency analysis of ERBB4 and SHBG SNP genotypes and alleles in PCOS

The research examined *ERBB4* and *SHBG* gene polymorphism genotypes and allele distribution patterns in PCOS women and their healthy age-matched counterparts based on the designed primers as depicted in Table 2 to determine their potential impact on PCOS development. The study used a case-control design that included equal numbers of cases and controls to calculate odds ratios with 95% confidence intervals under various genetic models that included allelic, homozygous, heterozygous, dominant, recessive and over-dominant models.

**Table 2.** Primer details for *ERBB4* gene polymorphisms (rs2178575 and rs1351592) and *SHBG* gene polymorphisms (rs1799941 and rs727428).

Gene (rsID)	Primers	Primer Sequence (5′ – 3′)	Melting Temperature (Tm)	PCR Product Size (bp)	Annealing Temperature
*ERBB4* rs2178575 G > A	Forward Outer	TCACATTTAACTTAGCCTGAGGGTAAAA	56	167	58.5^0^
	Reverse Outer	ATGTACTTTAAAGGAACCCTGCCAGT	56		
	Forward Inner (G allele)	AAATGATTAAAAATCCAGCGAGTCAAG	54	107	
	Reverse Inner (A allele)	TAAGGTCTTACCCCTTGAAATATAGCTGAT	58	117	
*ERBB4* rs1351592 C > G	Forward Outer	ATTGATAAGGACTACTGGCTAGAGTAGC	58	410	59.2^0^
	Reverse Outer	ATCCTCTTTATGGGTATTCTTCTTCTTC	56		
	Forward Inner (C allele)	GGTTTTTAGACAGAGTACATGTTAACACAC	58	215	
	Reverse Inner (G allele)	TTCAATTTCTTTTTAAATCCATCATACAC	52	254	
*SHBG* rs1799941 G > A	Forward Outer	TGTCCTTCACCCCATTGATCCCCAGAGG	64	314	68.4^0^
	Reverse Outer	GTGGGGAGAACAGGTCTCAGGGCCCATC	67		
	Forward Inner (G allele)	AACCTTTAACCCTCCACCGCCCACCCA	64	168	
	Reverse Inner (A allele)	GGAGAATGTGTAGAGGCAGGCAGCCTGGC	67	202	
*SHBG* rs727428 T > A	Forward Outer	AATGAGGACTTCTTAGGCTGAGATCCCA	60	330	64.6^0^
	Reverse Outer	CTCTGCTCTCCTCCCTTTTTCTTTCTCC	61		
	Forward Inner (T allele)	ATCAGGAGGAAGGGAAGAGATGGGAATA	60	208	
	Reverse Inner (A allele)	CCAAGGACCAGATGACTGAGATGTGAA	60	177	

All primer sequences are presented in the 5′ – 3′ orientation. Tm: Melting temperature; bp: base pairs.

ARMS-PCR was performed using allele-specific inner primers together with common outer primers for amplification of target polymorphisms.

The *ERBB4* rs2178575 polymorphism showed a significant relationship with PCOS risk through genotype-based studies and is depicted in Table 3. The homozygous model demonstrated that people with the AA genotype faced a higher PCOS risk than those with the GG genotype because the study found a significant chi-square result (χ^2^ = 17.47, *p* < 0.001) and an odds ratio that showed more than two times increased risk (OR = 2.48, 95% CI: 1.61–3.81).

**Table 3.** Genotypic and allelic distribution of *ERBB4* gene polymorphism rs2178575 among PCOS patients and controls.

Polymorphism	Frequencies	PCOS Patients (Cases) (*n* = 200)	Controls (*n* = 200)	OR	95% CI	χ^2^ / Fisher’s exact test	*p*-value
rs2178575	**Genotype**						
	GG	109	149	Reference			
	GA	4	3	1.823	0.400 − 8.307	0.702	0.496
	AA	87	48				
	**Allele**						
	A	178	99	2.438	1.805 − 3.293	34.464	<0.001
	G	222	301				
**Genetic Models**							
Homozygote (AA vs GG)	AA	87	48	2.478	1.611 − 3.810	17.466	<0.001
	GG	109	149				
Heterozygote (GA vs GG)	GA	4	3	1.823	0.40 − 8.25	0.464	0.496
	GG	109	149				
Over-dominant (GA vs GG + AA)	GA	4	3	1.340	0.296 − 6.066	1.000	0.317
	GG + AA	196	197				
Dominant (GA + AA vs GG)	GA + AA	91	51	2.439	1.599 − 3.722	17.469	<0.001
	GG	109	149				
Recessive (AA vs GA + GG)	AA	87	48	2.438	1.589 − 3.742	17.006	<0.001
	GA + GG	113	152				

* Odds ratios represent unadjusted estimates.

OR: Odds Ratio; CI: Confidence Interval. GG genotype and G allele were used as reference categories for odds ratio calculations. *p*-values were calculated using Chi-square test or Fisher’s exact test where appropriate. After Bonferroni correction (*p* < 0.002), only statistically significant associations retained significance.

The recessive model showed a significant association between AA and GA+GG with a χ^2^ value of 17.01 and a *p*-value below 0.001, which produced an OR of 2.44 with a 95% CI between 1.59 and 3.74 that showed the AA genotype increases PCOS risk. Neither the heterozygote (GA vs. GG) nor the over-dominant model (GA vs. GG+AA) was significantly associated with the disease status. This may be because of the rare occurrence of the GA genotype among both cases and controls. The dominance model (GA+AA vs. GG) also showed a significant association with PCOS risk; however, this is mainly because of the AA genotype and not the heterozygous GA genotype.

The allelic analysis of rs2178575 further supported the observed association. The A allele showed significant over-representation in PCOS cases compared to controls (χ^2^ = 34.46, *p* < 0.001), which demonstrated that it carried a two-fold increased risk of PCOS (OR = 2.44, 95% CI: 1.81–3.29). The rs2178575 polymorphism of *ERBB4* demonstrates a significant connection to PCOS susceptibility, with the effect predominantly driven by the A allele and AA genotype. Considering the low frequency of certain genotypes, particularly the GA genotype, the resulting wide CIs indicate limited precision of the estimates. Thus, alternative statistical approaches such as exact tests or penalized regression models may provide more stable estimates for the cases with low cell counts. This association remained statistically significant even after the Bonferroni correction for multiple comparisons. The gel image showing the PCR-amplified products is depicted in Figure S1.

The second *ERBB4* variant analysis showed that the distinct genetic pattern of rs1351592 created a strong link between this variant and PCOS and is represented in Table 4. The GC carriers who had the heterozygous model (GC vs GG) showed increased risk of developing PCOS because they demonstrated a statistical association χ^2^ = 5.29, *p* = 0.021, which resulted in an OR of 3.11 (95% CI: 1.14–8.50) that showed a strong heterozygote effect.

**Table 4.** Genotypic and allelic distribution of *ERBB4* gene polymorphism rs1351592 among PCOS patients and controls.

Polymorphism	Frequencies	PCOS Patients (Cases) (*n* = 200)	Controls (*n* = 200)	OR	95% CI	χ^2^ / Fisher’s exact test	*p*-value
rs1351592	**Genotype**						
	CC	90	137	Reference			
	CG	103	52	3.113	1.140 − 8.500	5.291	0.021
	GG	7	11				
	**Allele**						
	G	117	74	1.821	1.307 − 2.538	12.717	<0.001
	C	283	326				
**Genetic Models**							
Homozygote (GG vs CC)	GG	7	11	0.969	0.362 − 2.592	0.004	0.949
	CC	90	137				
Heterozygote (GC vs CC)	CG	103	52	3.015	1.968 − 4.619	26.473	<0.001
	CC	90	137				
Over-dominant (CG vs GG + CC)	CG	103	52	3.022	1.985 − 4.601	27.397	<0.001
	GG + CC	97	148				
Dominant (CG + GG vs CC)	CG + GG	110	63	2.658	1.767 − 3.998	22.500	<0.001
	CC	90	137				
Recessive (GG vs CG + CC)	GG	7	11	0.623	0.237 − 1.642	0.931	0.335
	CG + CC	193	189				

* Odds ratios represent unadjusted estimates.

OR: Odds Ratio; CI: Confidence Interval. CC genotype and C allele were used as reference categories for odds ratio calculations. *p*-values were calculated using Chi-square test or Fisher’s exact test where appropriate. After Bonferroni correction (*p* < 0.002), only associations meeting the corrected significance threshold were considered statistically significant.

The over-dominant model (GC vs. GG+CC) demonstrated strong results (χ^2^ value of 27.40 and a *p*-value below 0.001), with a three-fold increase in PCOS susceptibility (OR = 3.02; 95% CI:1.99–4.601), establishing that the GC genotype contributes to PCOS disease risk. The direct comparison of GC to CC through the co-dominant framework showed a strong association, which maintained its high significance level (OR = 3.02, 95% CI: 1.97–4.62). The recessive model (GG vs. GC+CC) did not show a statistically significant association, suggesting that homozygosity for the minor allele alone is insufficient to influence PCOS risk in this cohort. The G allele showed a significant link to PCOS, according to allelic analysis (χ^2^ = 12.72, *p* < 0.001; OR = 1.82, 95% CI: 1.31–2.54), which confirmed the genotype-based results. The study identified rs1351592 as a susceptibility locus through its allelic and heterozygous effects, while recessive inheritance did not prove to be a factor. This association after Bonferroni correction did not remain statistically significant and is therefore considered nominal. The gel image showing the PCR-amplified products is depicted in Figure S2.

The *SHBG* rs1799941 polymorphism showed a significant link to PCOS through its genotype distribution examination, according to the study results and is shown in Table 5. The cases showed a GA genotype excess, which reached 65.5% while the controls expressed it at a rate of 34.5%, and chi-square analysis confirmed a statistically significant difference (χ^2^ = 6.092, *p* = 0.014). The GA genotype carriers faced about double the risk when compared to GG homozygotes (OR = 2.091, 95% CI: 1.154–3.790). The research results showed identical findings through the over-dominant model, which compared GA against GG+AA, as it produced the same effect estimates, confirming its functioning through heterozygosity. The GG genotype produced a protective effect that reached statistical significance when compared with GA+AA (OR = 0.478, 95% CI: 0.264–0.867), supporting the observed association prior to multiple testing correction.

**Table 5.** Genotypic and allelic distribution of *SHBG* gene polymorphism rs1799941 among PCOS patients and controls.

Polymorphism	Frequencies	PCOS Patients (Cases) (*n* = 200)	Controls (*n* = 200)	OR	95% CI	χ^2^	*p*-value
rs1799941	**Genotype**						
	GG	164	181	Reference			
	GA	36	19	–	–	–	–
	AA	0	0				
	**Allele**						
	A	36	19	1.983	1.117 − 3.521	5.642	0.018
	G	364	381				
**Genetic Models**							
Heterozygote (GA vs GG)	GA	36	19	2.091	1.154 − 3.790	6.092	0.014
	GG	164	181				
Over-dominant (GA vs GG + AA)	GA	36	19	2.091	1.154 − 3.790	6.092	0.014
	GG + AA	164	181				
Dominant (GA + AA vs GG)	GA + AA	36	19	2.091	1.154 − 3.790	6.092	0.014
	GG	164	181				

* Odds ratios represent unadjusted estimates.

OR: Odds Ratio; CI: Confidence Interval. GG genotype and G allele were used as reference categories for odds ratio calculations. *p*-values were calculated using Chi-square test or Fisher’s exact test where appropriate. Homozygous and recessive model analyses could not be performed due to the absence of the AA genotype in both groups. After Bonferroni correction (*p* < 0.002), the observed associations were considered nominal and did not retain statistical significance.

The investigation received extra evidence through allelic analysis of the *SHBG* rs1799941 genetic variant. The A allele showed a significantly higher frequency in PCOS cases, and allelic association analysis confirmed this through statistical testing (χ^2^ = 5.642, *p* = 0.018). The A allele showed an approximate two-fold increase in PCOS risk (OR = 1.983, 95% CI: 1.117–3.521). The study did not benefit from recessive model analysis of this variant because both groups lacked the AA genotype; therefore, it was not investigated. This association should be interpreted with caution as the observed association results did not retain statistical significance after multiple testing correction. The gel image showing the PCR-amplified products is depicted in Figure S3.

The rs727428 polymorphism of the *SHBG* gene was studied to determine its relationship with PCOS risk. The study population showed a near-monomorphic genotype distribution, which demonstrated that T alleles were more common in both PCOS patients and control subjects. The analysis of recessive and heterozygote models became invalid because the study population showed extremely low occurrence of homozygous variants and heterozygous genotypes. The models failed to show any meaningful relationship with PCOS in their results.

The results show that genetic factors that affect PCOS susceptibility operate through specific loci and different models. The *ERBB4* rs2178575 variant displayed a risk pattern that followed a recessive and allelic distribution, while the *ERBB4* rs1351592 variant showed a strong impact from heterozygote carriers. *SHBG* rs1799941 established a connection to PCOS through its heterozygous state and its allelic inheritance pattern. The results demonstrate that PCOS has a complicated genetic structure, which includes various genes, and that different inheritance patterns must be assessed to study candidate gene links.

### 3.3. Minor allele frequency and Hardy-Weinberg equilibrium analysis

A summary of the minor allele frequencies (MAF) for the studied SNPs across the sample groups (both case and control) is presented in Table 6. The effective MAF for *ERBB4* rs2178575 was higher in cases (MAF = 0.445) than in controls (MAF = 0.2475), and in the same manner for rs1351592 (MAF = 0.2925 in cases, MAF = 0.185 in controls). The frequency of the minor allele of the polymorphism (rs1799941) of *SHBG* was low for both case (MAF =0.09) and control (MAF = 0.0475) sample groups.

**Table 6.** Minor allele frequency and Hardy-Weinberg equilibrium analysis of *ERBB4* and *SHBG* polymorphisms.

Gene Name	SNP	Chromosome	Base Change	Minor Allele Frequency			χ² (HWE in controls)	*p*-value
				Reference MAF (1000 Genomes – South Asian)	MAF Case	MAF Control		
*ERBB4*	rs2178575	chr2	G > A	0.194	0.445	0.2475	184.216	<0.0001
*ERBB4*	rs1351592	chr2	C > G	0.278	0.2925	0.185	3.7971	0.051
*SHBG*	rs1799941	Chr17	G > A	0.083	0.0.9	0.0475	0.4973	0.481

MAF: Minor Allele Frequency; HWE: Hardy–Weinberg Equilibrium. Control group genotype distributions were evaluated for HWE using the Chi-square goodness-of-fit test. Reference MAFs were obtained from the South Asian (SAS) population data of the 1000 Genomes Project. A *p*-value < 0.05 was considered indicative of deviation from Hardy–Weinberg equilibrium.

Comparison of the observed MAFs of the controls with data obtained from the 1000 Genomes Project of the South Asian population indicated that the observed frequencies of the controls were generally in agreement with the population frequencies observed (rs2178575 = 0.2475 vs 0.1940; rs1351592 = 0.185 vs 0.2780; rs1799941 = 0.0475 vs 0.0830), indicating there were no large variations in the distribution of the alleles.

The results of the Hardy–Weinberg equilibrium (HWE) analysis of the controls were that rs2178575 deviated significantly from HWE equilibrium (χ^2^= 184.216, *p* < 0.0001), rs1351592 had borderline significant deviation from HWE equilibrium (χ^2^=3.797, *p* = 0.051), and rs1799941 was in HWE equilibrium (χ^2^=0.497, *p* = 0.481).

### 3.4. Validation through Sanger sequencing

**Figure 1.**
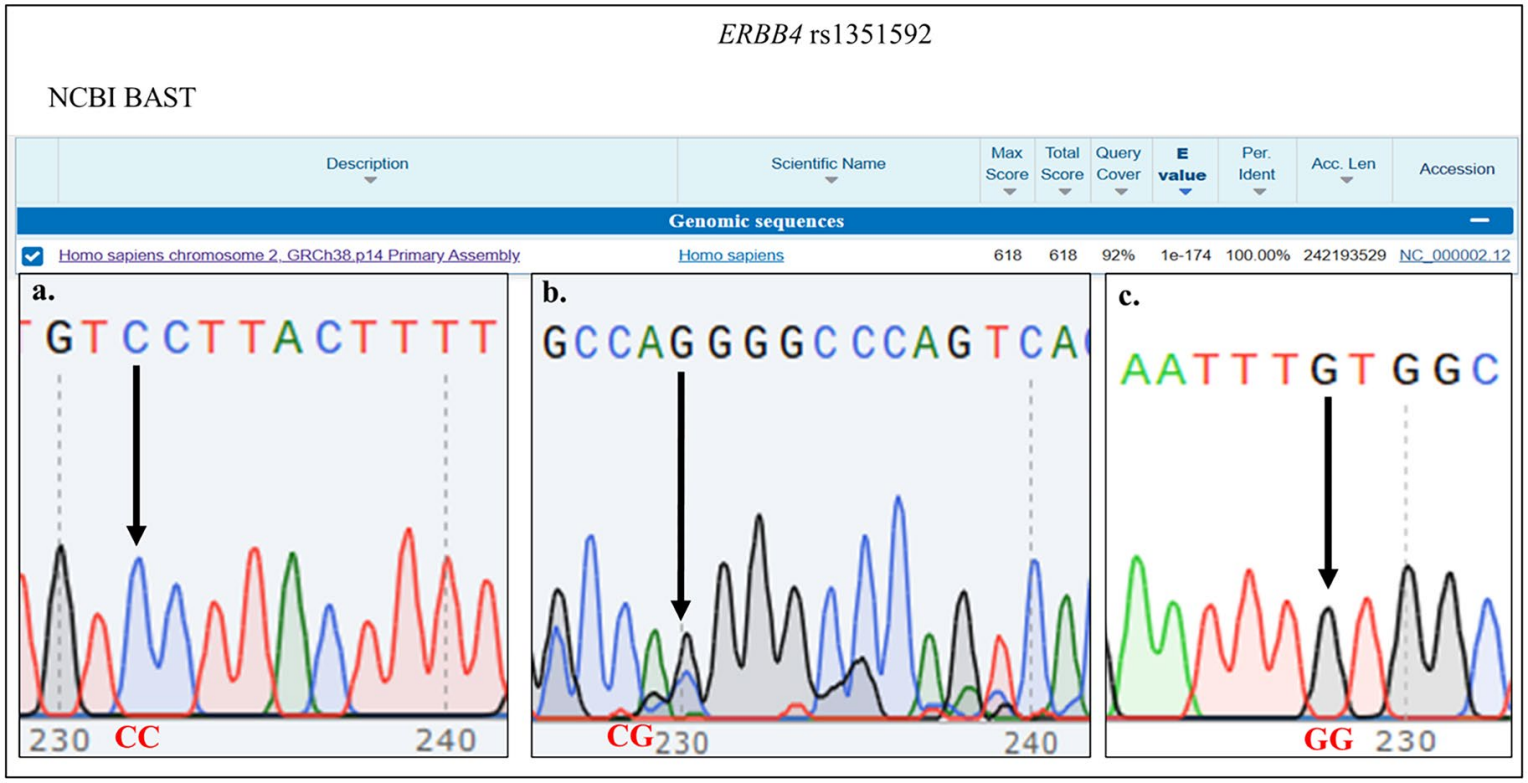
(a–c) Electropherogram results of Sanger sequencing for *ERBB4* gene (a) Homozygous wild-type (GG), (b) Heterozygous (GA), and (c) Homozygous mutant (AA) genotypes for rs2178575.

**Figure 2.**
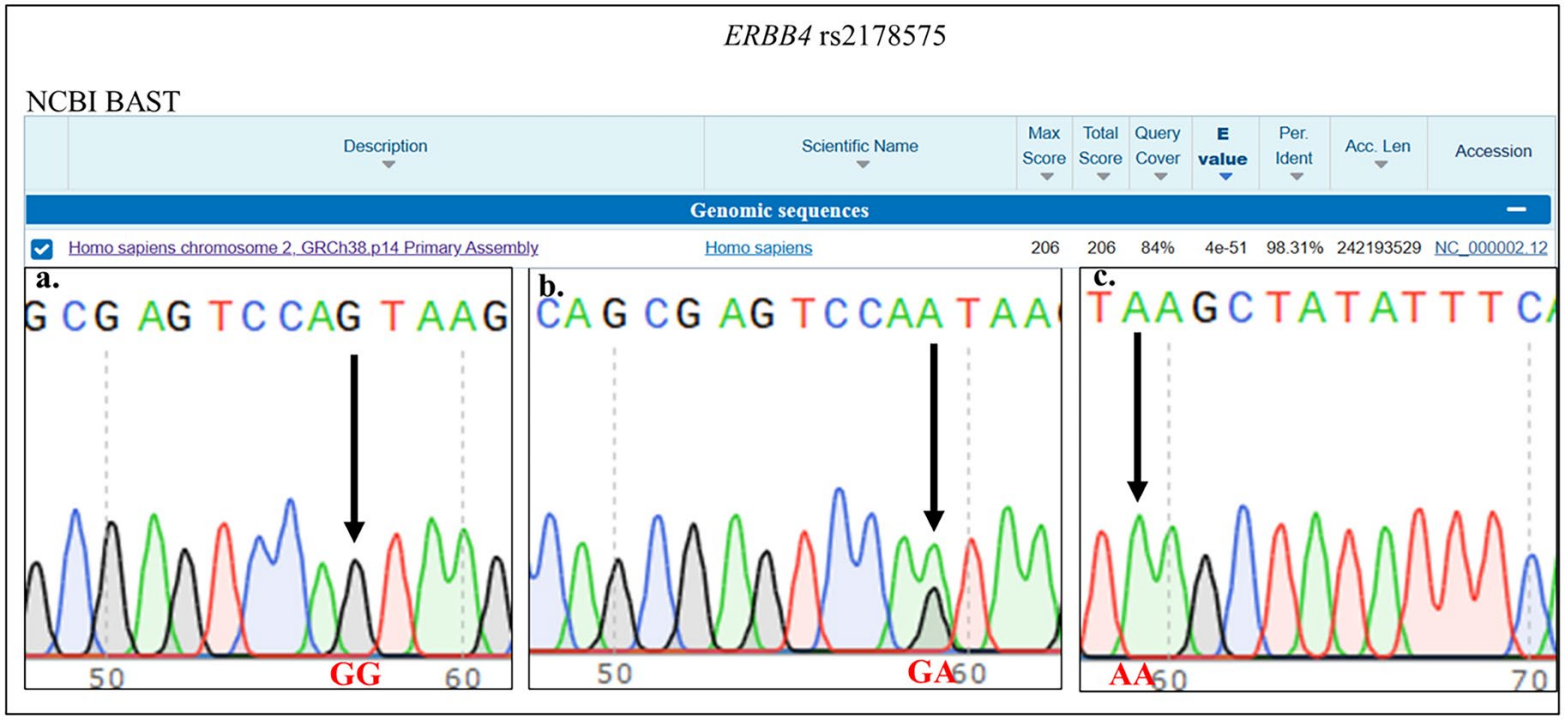
(a–c) Electropherogram results of Sanger sequencing for *ERBB4* gene (a) Homozygous wild-type (CC), (b) Heterozygous (CG), and (c) Homozygous mutant (GG) genotypes for rs1351592.

**Figure 3.**
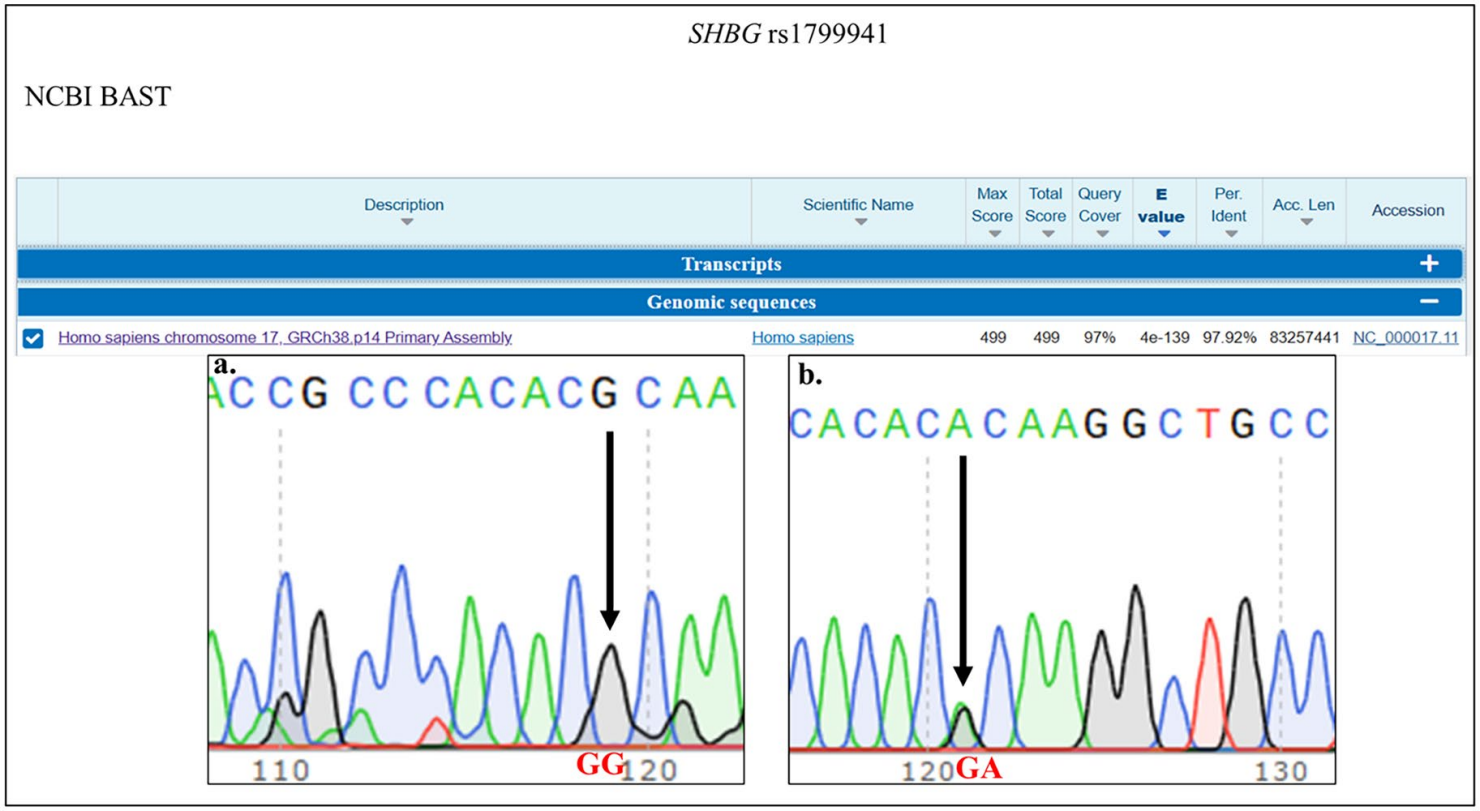
(a) and (b) Electropherogram results of Sanger sequencing for *SHBG* gene (a) Homozygous wild-type (GG), and (b) Heterozygous (GA) genotypes for rs1799941.

Sanger sequencing was employed to validate the genotypes obtained from the primary genotyping assays for rs2178575 (G > A) and rs1351592 (C > G) polymorphisms of the *ERBB4* gene and rs1799941 (G > A) polymorphism of the *SHBG* gene in a representative subset of PCOS cases and controls. Bidirectional sequencing was performed to ensure accuracy. The obtained electropherograms confirmed the presence of all three possible genotypes for both the SNPs of the *ERBB4* gene and two genotypes for the *SHBG* gene, which are depicted in Figure 1(a)–(c) for SNP rs2178575; Figure 2(a)–(c) for the SNP rs1351592 and Figure 3(a) and (b) for SNP rs1799941, thereby reinforcing the reliability of the genotypic data used in statistical and association analyses using NCBI BLAST.

## 4. Discussion

The resultant interplay of genetic, hormonal and metabolic factors is identified as the cause of PCOS, though recent research suggests the contribution of specific signalling and hormone-regulatory genes in the development of the condition. Despite GWAS studies aimed at understanding the major genetic variants involved in PCOS aetiology, the genetic mechanisms underlying disease susceptibility remain unclear [17]. The current study investigated PCOS susceptibility in association with two polymorphisms in the *ERBB4* gene (rs2178575 and rs1351592) and one polymorphism of the *SHBG* gene (rs1799941), employing a comprehensive model-based genetic approach. The evaluation of multiple inheritance models provided evidence associated with the locus-specific and model-dependent effects of PCOS risk, suggesting the heterogeneous nature of the disorder. Despite the fact that the current study is based on the findings of previous research into the relationship between *ERBB4* and *SHBG* polymorphism and PCOS, it offers further information by investigating several modes of inheritance among the South Indian population, an area that has been understudied in previous genetic research.

Results from the comparative analysis of the 1000 Genomes Project South Asian minor allele frequencies support the validity of the current study’s participant group. The observed control group genotype frequencies were generally consistent with the reference, indicating neither significant population stratification occurred nor bias due to the use of self-reported South Indian ethnicity as ascribed by participants in this study. Additionally, while allele frequencies matched well to those reported for South Asians, the questioning about the presence of other sub-ethnic/ethno-cultural groups on population heterogeneity cannot be excluded. Future studies including exploration using genome-wide markers (e.g. other South Asian datasets) as well as multi-dimensional PCA will be necessary to better assess the structure of these populations. Analyses of the HWE at the rs2178575 locus in the study sample’s control group demonstrated deviation from HWE. Such a deviation is likely due to the relatively few heterozygotes at this locus and may be attributable to sampling variation, substructure present within the population, and potential genotyping biases. Both loci (i.e. rs1351592 and rs1799941) were also determined to be in HWE, which supports the reliability of the observed genotype data collected for these loci across all participants. Additionally, while rs1799941 showed the greatest number of low-frequency observed genotypes amongst all three loci described above, any such low-frequency genotype by itself represents a potential limitation of the statistical power associated with any individual study end-points involving those loci.

The present study identified a significant association between the SNP rs2178575 of the *ERBB4* gene and PCOS risk, particularly under allelic and recessive genetic models. The observed allele-dosage effect, reflected by the enrichment of the A allele and AA genotype among the PCOS cases, suggests a potential role of this polymorphism in disease susceptibility. This finding was in concordance with the results of previous studies that suggest the established role of the *ERBB4* gene in the production of androgen and ovarian physiology, regulating follicular growth, oocyte maturation and granulosa cell differentiation. The epidermal growth factor receptor family contains *ERBB4,* which functions as a vital component for cellular signalling through PI3K, AKT and MAPK signalling pathways. The pathways control both cell growth and development while they also manage metabolic processes which involve glucose absorption and insulin response and the maintenance of cellular energy balance. The dysregulation of *ERBB4*-mediated signalling establishes a connection between two main PCOS symptoms, which are impaired folliculogenesis and metabolic disturbances that include insulin resistance. The genetic variations in the *ERBB4* gene cause changes to these signalling mechanisms, which result in ovarian dysfunction and an increased risk of developing PCOS [13]. The study conducted by Samma et al. (2024) showed a similar significant association between the rs2178575 polymorphism of the *ERBB4* gene among the infertile PCOS patients based on the chi-square analysis (χ^2^ = 10.282, *p* = 0.005852). Their results further showed a reduced risk of infertility among the individuals having the heterozygous (GA) and mutant (AA) genotype with OR = 0.541 in comparison with the wild-type (GG) genotype, indicating a protective genetic effect of the polymorphism among the females in Pakistan [18]. Thus, these findings suggest *ERBB4* rs2178575 as a potential genetic contributor to PCOS susceptibility while modulating the risk of infertility. Due to the moderate sample size and lack of functional validation, the observed associations should be interpreted with caution.

Conversely, the heterozygous and over-dominant models demonstrated a significant association in the *ERBB4* rs1351592 gene polymorphism compared to the recessive model, illustrating a heterozygote-driven pattern of association in the present study. This kind of heterozygote-specific effect was shown to be increasingly high in endocrine disorders, exhibiting more substantial phenotypic consequences than the homozygous conditions [19]. A study conducted by Peng et al. (2017) confers the risk of PCOS with OR = 1.310 (95%CI: 1.114–1.540) among the Han Chinese population carrying the G allele, suggesting a pivotal role of *ERBB4* in the susceptibility of PCOS across the population [20]. In contrast, the study conducted by Afzal et al. (2021) among the females of Pakistan showed a protective trend of *ERBB4* gene polymorphism, although insignificant, with OR = 0.69 (95% CI: 0.32–1.53, *p* = 0.37) [14]. These observed results exhibit the complex and ethnic variability of PCOS susceptibility in association with the heterozygous nature of the *ERBB4* gene polymorphism. Such inconsistencies across populations may be attributed to differences in genetic background, allele frequency distributions and environmental factors, mainly emphasizing the importance of population-specific studies.

The contribution of hormonal regulatory genes in the pathogenesis of PCOS is further supported by the association of the *SHBG* rs1799941 gene polymorphism in the current study. A two-fold increased risk of the GA genotype in comparison with the GG genotype has been observed in the present study among the cases. The role of heterozygosity at this particular locus was confirmed based on the consistent results obtained for the over-dominant model. Whereas the protective role was exhibited by the GG genotype, revealing the robustness and bidirectional nature of this association. Due to the absence of AA homozygotes in both cases and controls, the recessive model analysis was not carried out. *SHBG* functions as the primary regulator for sex steroid availability because it binds both circulating androgens and estrogens, which results in controlling their active unbound forms. The relationship between *SHBG* and insulin sensitivity exists because hyperinsulinemia results in decreased hepatic SHBG production, which leads to higher free androgen levels. This condition produces essential symptoms of PCOS, which include hyperandrogenism and ovulatory dysfunction together with metabolic irregularities. The decrease in *SHBG* results in higher percentages of free androgen levels, which intensifies hirsutism, acne and ovulatory disorders. Consequently, genetic variants in the *SHBG* gene may influence PCOS risk not only through hormonal imbalance but also through modulation of insulin resistance and metabolic homeostasis which also impact insulin resistance and metabolic characteristics that directly relate to PCOS disease progression [21,22].

Previous studies involving the metabolic regulatory genes such as *TCFL2* have also been shown to have an impact on PCOS pathogenesis due to their role in insulin secretion and glucose homeostasis, showing a shared genetic basis between PCOS and type-2 diabetes-related traits [23]. To our knowledge, however, the only other Mendelian randomization study to date was that by Guo et al. (2024), and they found that genetically instrumented higher circulating SHBG concentrations were associated with a lower risk of PCOS, indicating a protective role for *SHBG*. This seems to contradict the current results in which an increased risk of PCOS was found in carriers of the GA genotype of rs1799941. However, this difference might be attributed to the fact that genetic polymorphisms are not always a direct translation of circulating protein level or functional activity. The rs1799941 variant might affect *SHBG* regulation by multifactorial mechanisms acting at transcriptional or post-transcriptional levels, and such associations may be population-specific [24]. Thus, in the present study we mainly focus on the genetic variation at rs1799941, which shows a potential association with PCOS vulnerability, establishing a biological connection between genetic differences, hormonal disorders and disease development. Conversely, the study conducted by William et al. (2011) found no significant association for the rs1799941 gene polymorphism among the PCOS patients in Pennsylvania [25]. Similar results were shown by Martínez-García and colleagues among the Mediterranean population [26]. These findings, however, require further validation in larger and ethnically diverse populations.

Collectively, both *ERBB4* and *SHBG* play interconnected roles linking reproductive and metabolic pathways in PCOS. The *ERBB4* gene is essential in the development of the follicle and for the interactions between the oocyte and granulosa cells due to its function within the epidermal growth factor signalling pathway. Variations within this gene may affect the signalling pathway, resulting in defective folliculogenesis and anovulation. Likewise, the SHBG hormone controls the bioavailability of the circulating androgens, and variations may result in hyperandrogenism, which is a key feature of PCOS.These interaction among the pathways shows the dual endocrine-metabolic nature of PCOS, where ovarian function and metabolic homeostasis are affected by the genetic variations.

The research findings demonstrate that genetic factors that operate through defined genomic regions and inheritance methods establish PCOS susceptibility. The *ERBB4* rs2178575 polymorphism has shown association in the recessive and allelic genetic models, while rs1351592 demonstrates a strong impact on heterozygote carriers (GC genotype). The *SHBG* rs1799941 genetic variant leads to PCOS risk through two primary ways, which include heterozygosity and allelic inheritance. The combined genetic effects could not be evaluated through haplotype or LD analysis for both *ERBB4* and *SHBG* genes, as the distinct association patterns observed suggest that these variants may act independently in influencing PCOS susceptibility. The research results demonstrate that PCOS genetic variation exists in multiple forms, which need assessment through different genetic testing methods. However, while there was a noticeable difference in the clinical and biochemical markers between women with PCOS and the control group, no genotype-phenotype correlation studies were conducted. As such, the exact association between the polymorphisms tested and the clinical presentation, including BMI, LH/FSH ratios, and testosterone concentrations, is unknown. BMI can be considered a confounding factor as well as an intermediate variable in the relationship between polymorphisms and PCOS, since there is a strong correlation between BMI and metabolic and hormonal characteristics of PCOS. Hence, the lack of a BMI-adjusted analysis can impact the findings. The interpretation of the findings is substantiated by the magnitude and precision of the estimated odds ratios. Nevertheless, the larger confidence intervals in some genetic models, especially those with rare genotypes, imply low statistical precision. Although the research helps to gain an understanding of the genetics of PCOS in South Indians, the results must be considered in light of the methodology employed in the study.

### 4.1. Limitations

However, several issues related to the current study must be highlighted despite the significant associations observed. First, the size of the sample, which is adequate for identifying strong genetic links, may not be sufficient to draw definitive conclusions. Second, the stringent matching factors such as BMI and reproductive parameters like parity were not fully controlled, which might affect the associations found. Furthermore, no extensive population stratification analysis was conducted, which may introduce bias. It is significant to note that the current study does not incorporate any functional tests aimed at assessing the influence of the detected genetic polymorphisms. There was no investigation into the effect of *ERBB4* or *SHBG* polymorphisms on gene expression at the mRNA level and protein expression; thus, there is a lack of functional validation of the observed genetic associations. Additionally, there were no correlation studies to examine the association between genetic polymorphisms and circulating biomarkers such as serum ERBB4 concentration or SHBG levels. Additionally, stratified analyses based on testosterone levels were not performed, which may have provided further insights into genotype-phenotype associations. The present study did not explore haplotype structures or gene-gene interactions and as well as LD analysis between *ERBB4* and *SHBG* variants, which may provide deeper insights into the combined genetic effects underlying PCOS susceptibility. Selection bias could be caused by recruiting cases and controls from different hospitals, although all patients followed identical inclusion/exclusion criteria. Furthermore, since controls were recruited from only one hospital, this limits the generalizability of the findings.

## 5. Conclusion

The results of the current study indicated that the *ERBB4* and *SHBG* gene polymorphisms might be potential risk factors for PCOS in South Indian women. *ERBB4* rs2178575 showed a strong association in allelic and recessive models, suggesting a genotype-driven effect, whereas rs1351592 showed a heterozygote-specific association. *SHBG* rs1799941 polymorphism was also associated with PCOS in heterozygous and allelic models, whereas rs727428 was not in this population group. Taken together, these results suggest that different genetic variants influence risk for PCOS through different modes of inheritance, indicating a complex genetic basis for this disorder. Further studies involving larger, well-characterized cohorts, stringent participant matching, and functional validation are required to confirm these findings and elucidate underlying molecular mechanisms.

## Supplementary Material

Supplementary File.docx

## Data Availability

The datasets generated and analyzed during the current study are not publicly available due to institutional restrictions, but are available from the corresponding author on reasonable request.

## Abbreviations

PCOS: Polycystic ovarian syndrome
*ERBB4* gene: erb-b2 receptor tyrosine kinase 4
*SHBG*: Sex hormone–binding globulin
OR: odds ratio
CI: confidence interval
HWE: Hardy–Weinberg equilibrium
ARMS-PCR: Amplification Refractory Mutation System Polymerase Chain Reaction
