# Supplementary material for: Association of ERBB4 and SHBG gene polymorphisms with polycystic ovarian syndrome in South Indian women: a case–control genetic analysis
Source: Ann Med. 2026 Jun 18;58(1):2688583. doi: 10.1080/07853890.2026.2688583 (PMC13312336; doi:10.1080/07853890.2026.2688583)
Supplement: Supplementary File.docx [file IANN_A_2688583_SM8847.docx]

**Figure Legends**

**Supplementary Figure S1:** Agarose gel electrophoresis image showing PCR amplification products of the *ERBB4* gene for SNP rs2178575 genotyping. Lane 1: 100 bp DNA ladder. Lane 2: GG genotype showing the common outer band (167 bp) and G allele-specific band (107 bp). Lane 13: AA genotype showing the common outer band and A allele-specific band (117 bp). Lane 17: GA heterozygous genotype showing both allele-specific bands.

**Supplementary Figure S2:** Agarose gel electrophoresis image showing PCR amplification products of the *ERBB4* gene for SNP rs1351592 genotyping. Lane 1: 100 bp DNA ladder. Lane 4: GG genotype showing the common outer band (410 bp) and G allele-specific band (254 bp). Lane 8: CG heterozygous genotype showing both allele-specific bands. Lane 9: CC genotype showing the common outer band and C allele-specific band (215 bp).

**Supplementary Figure S3:** Agarose gel electrophoresis image showing PCR amplification products of the *SHBG* gene for SNP rs1799941 genotyping. Lane 1: 100 bp DNA ladder. Lane 2: GG genotype showing the common outer band (314 bp) and G allele-specific band (168 bp). Lane 7: GA heterozygous genotype showing both G allele-specific (168 bp) and A allele-specific (202 bp) bands.

**
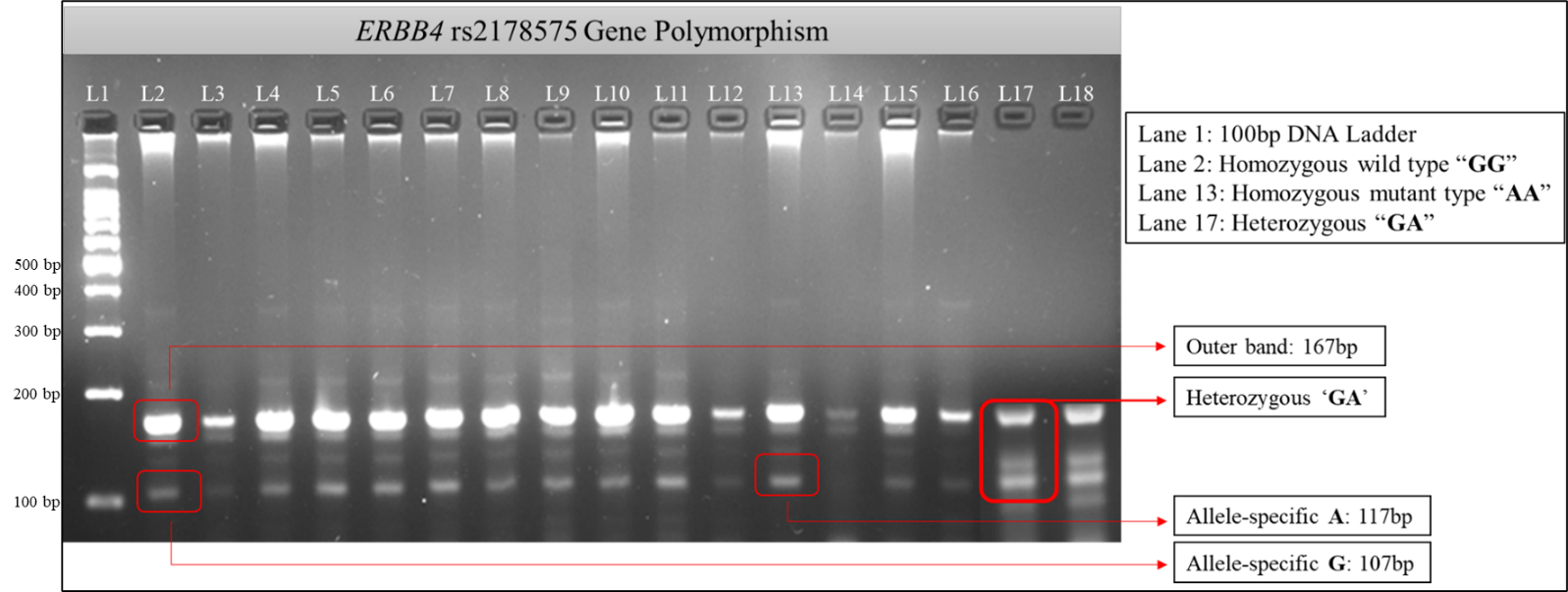
Supplementary Figure S1:** Agarose gel electrophoresis image showing PCR amplification products of the *ERBB4* gene for SNP rs2178575 genotyping.


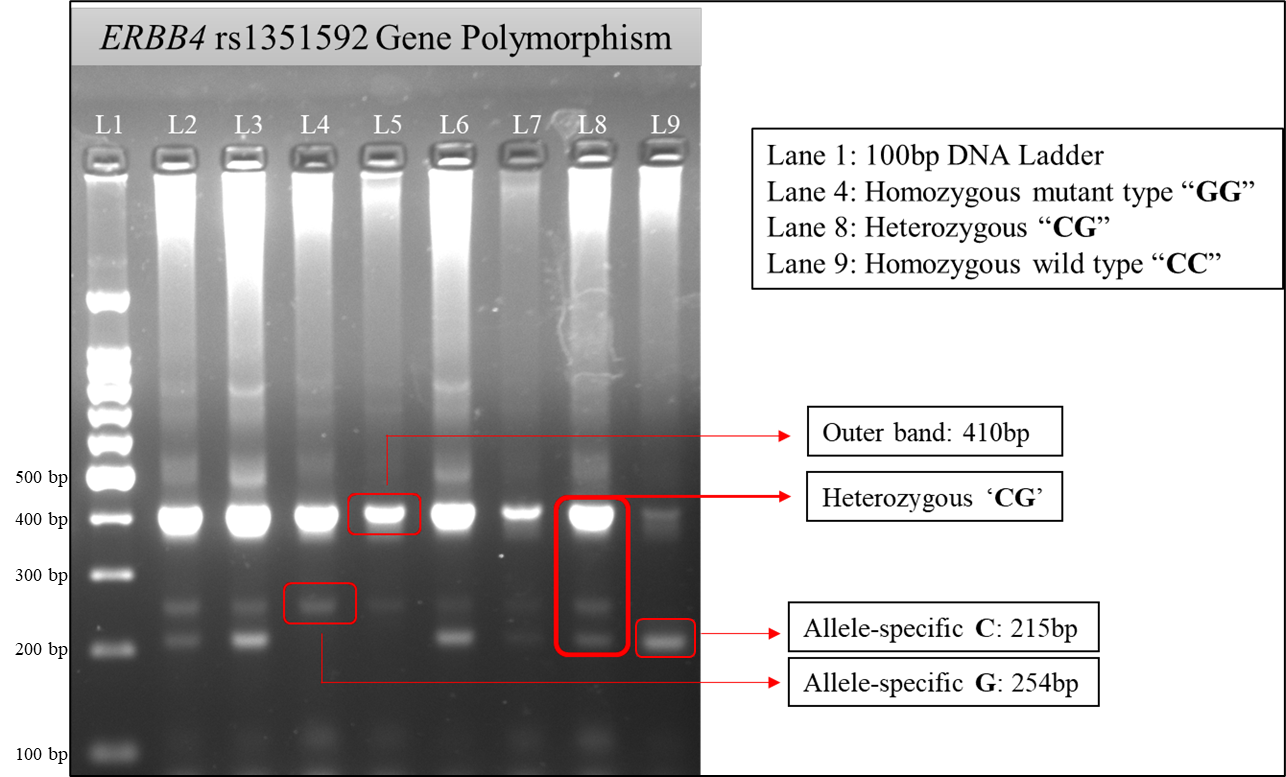


**Supplementary Figure S2:** Agarose gel electrophoresis image showing PCR amplification products of the *ERBB4* gene for SNP rs1351592 genotyping.

**
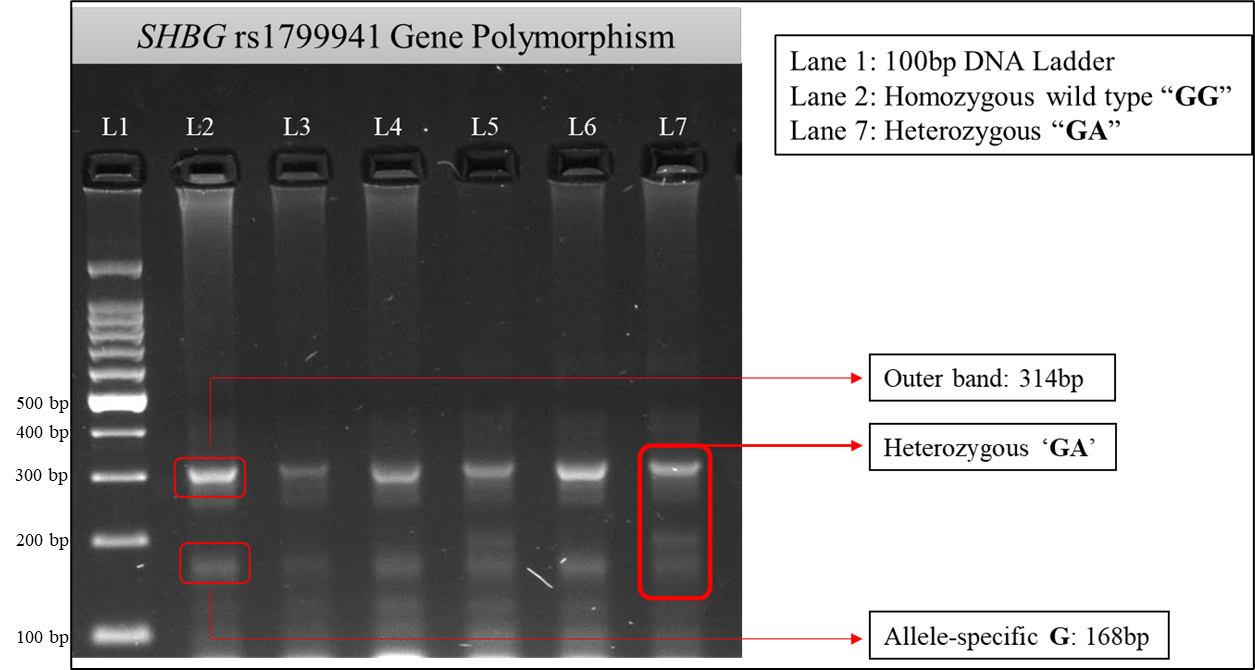
Supplementary Figure S3:** Agarose gel electrophoresis image showing PCR amplification products of the *SHBG* gene for SNP rs1799941 genotyping.
